# Conducting Research in Persons With Dementia During the COVID-19 Pandemic: Challenges and Strategies

**DOI:** 10.1093/geroni/igab046.1201

**Published:** 2021-12-17

**Authors:** Ying-Ling Jao, Diane Berish, Ann Kolanowski

## Abstract

The COVID-19 pandemic has caused a health crisis for vulnerable older adults, physically and psychologically. Despite the urgent demand for clinical research for people with dementia, research activities are restricted due to the pandemic. This symposium will share the experiences of researchers conducting studies with older persons with dementia during the COVID-19 pandemic. The presenters share their strategies to overcome challenges at different stages of the study process during the pandemic. The research projects include work conducted in acute care, assisted living, nursing homes, and the community. The presentations include perspectives from different geographic areas and across countries in North America. The first presenter reports the challenges in continuing an ongoing research project, and shares strategies to engage stakeholders and plan a new protocol for recruitment and in-person data collection with residents with dementia in nursing homes. The second presenter reports on the barriers and facilitators of conducting an ongoing clinical trial with older adults with dementia across hospital and community settings and discusses strategies to meet project goals which include modifications to the protocol and analytic plan. The third presenter describes adaptations made to a study intervention designed to promote quality resident-staff interactions in assisted living and alterations to stakeholder engagement. The fourth presenter describes challenges and strategies to engage older adults with dementia via technology. The discussant will synthesize the findings across studies and highlight policy and research implications for the COVID-19 pandemic as well as other emergency situations.

